# Degradation of cochlear Connexin26 accelerate the development of age‐related hearing loss

**DOI:** 10.1111/acel.13973

**Published:** 2023-09-08

**Authors:** Kai Xu, Sen Chen, Xue Bai, Le Xie, Yue Qiu, Xiao‐zhou Liu, Xiao‐hui Wang, Wei‐jia Kong, Yu Sun

**Affiliations:** ^1^ Department of Otorhinolaryngology, Union Hospital, Tongji Medical College Huazhong University of Science and Technology Wuhan China; ^2^ Hubei Province Key Laboratory of Oral and Maxillofacial Development and Regeneration Wuhan China

**Keywords:** age‐related hearing loss, Connexin26, hair cells, spiral ganglion neurons

## Abstract

The GJB2 gene, encoding Connexin26 (Cx26), is one of the most common causes of inherited deafness. Clinically, mutations in GJB2 cause congenital deafness or late‐onset progressive hearing loss. Recently, it has been reported that Cx26 haploid deficiency accelerates the development of age‐related hearing loss (ARHL). However, the roles of cochlear Cx26 in the hearing function of aged animals remain unclear. In this study, we revealed that the Cx26 expression was significantly reduced in the cochleae of aged mice, and further explored the underlying molecular mechanism for Cx26 degradation. Immunofluorescence co‐localization results showed that Cx26 was internalized and degraded by lysosomes, which might be one of the important ways for Cx26 degradation in the cochlea of aged mice. Currently, whether the degradation of Cx26 in the cochlea leads directly to ARHL, as well as the mechanism of Cx26 degradation‐related hearing loss are still unclear. To address these questions, we generated mice with Cx26 knockout in the adult cochlea as a model for the natural degradation of Cx26. Auditory brainstem response (ABR) results showed that Cx26 knockout mice exhibited high‐frequency hearing loss, which gradually progressed over time. Pathological examination also revealed the degeneration of hair cells and spiral ganglions, which is similar to the phenotype of ARHL. In summary, our findings suggest that degradation of Cx26 in the cochlea accelerates the occurrence of ARHL, which may be a novel mechanism of ARHL.

## Graphical abstract

In this paper, our results support the hypothesis that reduction of Cx26 and disruption of GJPs in the cochlea contribute to the development and progression of age‐related hearing loss. The degradation of Cx26 tends to occur in the early stages of age‐related hearing loss. Therefore, it can be used as an early warning marker of age‐related hearing loss.

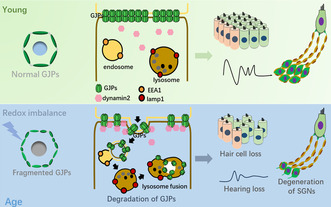

**Abbreviations**
ABR: auditory brainstem response
ARHL: age‐related hearing loss
Cx26: Connexin26
DCs: deiters' cells
Dyn2: dynamin 2
ER: endoplasmic reticulum
GJPs: gap junction plaques
GO: glucose oxidase
GWAS: genome‐wide association studies
IHC: inner hair cell
OHCs: outer hair cells

## 1 INTRODUCTION

With the increased aging of the global population, the prevalence of age‐related diseases continues to rise. Age‐related diseases have received more and more attention, but they remain a worldwide problem (Dzau & Balatbat, 2018). Age‐related hearing loss (ARHL), also known as presbycusis, is the most common chronic sensory disorder in older individuals. The clinical characteristics of ARHL are bilateral progressive high‐frequency hearing loss with age, and accompanied poor speech discrimination (Davis et al., 2016). The prevalence of ARHL increases with age, affecting approximately 40% of people over 50 years old (Slade et al., 2020). Hearing loss may lead to a reduction in social interaction, increased loneliness and depression, and ultimately cognitive decline and dementia in older adults (Jafari et al., 2021; Uchida et al., 2019). The development of ARHL is typically influenced by various factors, including genetic variants, environmental factors, and co‐regulation (Wells et al., 2020). Currently, our understanding of the pathogenesis of ARHL is limited, and the use of hearing aids and cochlear implants is the means of helping ARHL patients improve their hearing. Therefore, there is an urgent need to further explore the underlying pathogenesis and formulate mechanism‐based treatment.

For the prevention or early intervention of ARHL, understanding the etiology, pathogenesis and risk factors of this disease is crucial. Currently, there is increasing evidence linking certain genes to the development of ARHL (Lewis et al., 2018; Van Laer et al., 2010). Several ARHL genome‐wide association studies (GWAS) have been carried out and have identified multiple candidate genes or loci associated with adult‐onset hearing loss (Friedman et al., 2009; Hoffmann et al., 2016; Vuckovic et al., 2015). Rodriguez‐Paris et al. (2008) reported that carrying two mild mutations in the GJB2 gene may increase the risk of developing early presbycusis. However, adult‐onset hearing loss may result from single genetic variants or the additive effect of multiple gene variants with minor effects. Even different variations in the same gene can lead to multiple auditory phenotypes. In addition to endogenous genetic factors, ARHL is considered to be a disease with multiple extrinsic potential risk factors, including environmental factors (e.g., noise exposure, ototoxic drug use) and health comorbidities (e.g., hypertension, diabetes) (Fetoni et al., 2022; Samocha‐Bonet et al., 2021; Wang & Puel, 2020; Yamasoba et al., 2013). Noise exposure and ototoxic drugs are both independent factors causing hearing loss and external factors aggravating the progression of ARHL (Yang et al., 2015). A comprehensive cohort study has revealed that Type 2 diabetes is associated with the incidence of hearing loss in the older population. Moreover, the progression of hearing loss is significantly accelerated in patients with newly diagnosed diabetes (Mitchell et al., 2009). However, there is still a subset of patients with age‐related deafness who neither carry known causative mutations nor have been exposed to the known external and internal factors, suggesting that there are still unknown universal age‐related deafness mechanisms that have not been elucidated.

A number of genes involved in the development of inner ear are known to be essential for normal hearing development and maintenance of inner ear homeostasis. Mutations in a gene‐related deafness lead to changes in the function of the protein it encodes, resulting in deafness. In addition, abnormal protein synthesis and degradation caused by changes in epigenetic regulation or physiological state can also lead to impaired hearing function (Kuo et al., 2021; Pouyo et al., 2021). In recent years, researchers have focused on the link between ARHL and changes in various of deafness‐related genes and their encoded proteins. Mutations of the GJB2 gene cause various auditory phenotypes, including congenital deafness or late‐onset progressive hearing loss (Chan & Chang, 2014; Orzan & Murgia, 2007). This diversity of auditory phenotypes is due to different types of mutations in the GJB2 gene. The common genotypes 35delG and 235delC cause truncation mutations that result in severe to profound sensorineural hearing loss (Fuse et al., 1999; Kelley et al., 1998; Morell et al., 1998). The V37I (c.109G > A) variant of GJB2 has a high prevalence (up to 10%) in East Asians. Both homozygous p.V37I variants and compound heterozygous p.V37I with other GJB2 pathogenic variants result in mild to moderate hearing loss (Shen et al., 2017). A variety of Cx26 conditional knockout mouse models also showed a phenotype ranging from mild to moderate to severe hearing loss (Chen, Xu, et al., 2018; Sun et al., 2009; Zhu et al., 2015). Connexins have been identified in a variety of tissues with short half‐lives of 1–5 h (Falk et al., 2009, 2014). The gap junctions on the cell membrane are constantly renewed, and old connexin is continuously replaced by newly synthesized connexins (Carette et al., 2015; Piehl et al., 2007). The synthesis and degradation of connexins are in equilibrium under physiological conditions. However, under pathological conditions, the balance between synthesis and degradation of connexins may be broken. Recently, several studies have shown an association between Cx26 and ARHL. Ichimiya et al. (2000) first reported that expression of Cx26 was significantly reduced in the spiral ligament of aged mice. Our previous studies have reported that Cx26 expression and hypermethylation of its DNA promoter were both decreased in the inner ear of mimetic aging rats (Wu et al., 2014). Fetoni et al. (2018) constructed a Gjb2^+/−^ mouse model to simulate the heterozygous population carrying Gjb2 gene mutations and found that Cx26 partial loss causes accelerated presbycusis by inducing redox imbalance and dysregulation of the Nfr2 pathway. Tajima et al. (2020) also demonstrated that degradation of Cx26 and disruption of gap junction plaques are involved in the early development of ARHL. Although these data suggest that gap junction dysfunction may be involved in the development of presbycusis, it is not sufficient to prove that Cx26 is an independent risk factor for presbycusis. The underlying mechanism of cochlea gap junction degradation and its role in the etiopathogenesis of ARHL needs to be further explored.

In this study, we investigated the changes of Cx26 expression and gap junction plaques in the cochlea with aging. In the cochleae of aged mice, Cx26 expression levels decreased and gap junction plaques (GJPs) appeared drastically fragmented. Fragmented GJPs are internalized into the cytosol and eventually degraded in lysosomes. Furthermore, we generated a mouse model of cochlea Cx26‐knockout in juvenile mice to simulate age‐related Cx26 degradation and analyze the auditory phenotype. The knockout mice showed specific high‐frequency hearing loss and hair cell death in the basal turn, which is similar to the phenotype of ARHL.

## 2 MATERIALS AND METHODS

### 2.1 Mouse models

Male 4‐week‐old or 13‐month‐old C57/6J mice were obtained from the experimental animal center of Tongji Medical College, Huazhong University of Science and Technology. Tamoxifen‐inducible Cx26‐null mice were generated by crossbreeding of the Cx26^f/f^ mice with Sox2‐CreERT2 or Fgfr3‐iCreERT2 mice. All mice were injected with tamoxifen (T5648‐1G, Sigma–Aldrich) subcutaneously at P17 and P18 (the total dose was 1.5 mg/10 g body weight). Details of the identification of mouse genotyping were as described in previous studies (Xu et al., 2020; Zhang et al., 2020).

### 2.2 Auditory brainstem response (ABR)

The hearing thresholds were measured by ABR at P50 or P80. Tucker‐Davis Technology (TDT) System was used to measure the hearing threshold as previously publication (Chen et al., 2014). Briefly, after anesthetized and the mice were put on the 37°C thermostatic electric blanket. The recording electrode and reference electrode were inserted under the skin of the skull or the tested ear, respectively. Six frequencies (4, 8, 16, 24, 32, and 40 kHz) were tested, and the SigGen32 software (Tucker‐Davis Technologies) was used to amplify and record the ABR signals at different frequencies.

### 2.3 Protein extraction and western blotting

Total protein was extracted from the cochleae using RIPA lysis buffer (P0013B, Beyotime). Samples containing equal amounts of protein were separated by 12% sodium dodecyl sulfate–polyacrylamide gel electrophoresis (SDS‐PAGE) and then transferred to polyvinylidene difluoride (PVDF) membranes. After blocking in tris‐buffered saline (TBST with 0.1% Tween‐20) containing 5% milk for 1.5 h, the Cx26 and β‐actin proteins were detected using anti‐Cx26 antibody (710500, Invitrogen) or anti‐β‐actin antibody (04‐1116, Merck‐Millipore). Then the PVDF membranes were incubated with horseradish peroxidase‐conjugated secondary antibody, the bands were visualized using an ECL reaction kit and the intensities were semi‐quantified.

### 2.4 RNA preparation and real‐time quantitative polymerase chain reaction

Details of RT‐qPCR have been described in our previous study (Xu et al., 2020). Briefly, Total RNA was extracted from whole cochleae with Trizol Reagent and then reverse transcribed by using PrimeScript RT reagent kit with gDNA eraser (Takara Bio Inc.). The qRT‐PCR was performed on a LightCycler™ 480 system (Roche Diagnostics Ltd) with the LightCycler 480 SYBR Green I Master. The mRNA level was normalized to GAPDH, and the results were analysis with the comparative cycle threshold 2^−ΔΔCP^ method.

### 2.5 Cochlear tissue preparation and immunofluorescent labeling

The acquisition of cochlear specimens has been detailed in our previous study (Chen, Xu, et al., 2018). The samples were permeabilized with 0.3% Triton X‐100 for 20 min. After blocking with 5% bovine serum albumin for 1 h, then incubated with anti‐Cx26 antibody (710500, Invitrogen), anti‐EEA1 antibody (ab2900, Abcam), anti‐LAMP1 antibody (ab25245, Abcam), anti‐Dynamin 2 antibody (14605‐1‐AP, Proteintech), and anti‐myosin 7a antibody (25‐6790, Proteus Bio‐Sciences). Then the sample were washed with PBS and incubated with secondary antibody for 2 h at room temperature. The samples were counterstained with DAPI to label the nucleus and then observed with laser‐scanning confocal microscope (Nikon).

### 2.6 Cochlear explant culture

Wild‐type C57BL/6 mice were sacrificed at P3, and the cochlear basilar membrane was carefully isolated and placed in cooled PBS, then the cochlear explants were placed on a pre‐prepared collagen gel matrix. Basal medium consisting of bovine serum albumin (A8020, Solarbio), glutamine (G7513, Sigma‐Aldrich), glucose and penicillin G (P3414, Sigma‐Aldrich) was added, and the culture dish was transferred to an incubator at 37°C with 5% CO2 overnight before each treatment. On the following day, cochlear explants were treated with different concentrations of hydrogen peroxide or GO for 4 h, and harvested for hair cell counting.

### 2.7 Transmission electron microscopy

Samples were fixed in a mixture of 2% paraformaldehyde and 2.5% glutaraldehyde (Sigma‐Aldrich, G5882), then decalcified for 48 h in 10% disodium EDTA and immobilized in 1% osmium tetroxide (Sigma‐Aldrich, O5500). After dehydrating with acetone, then the sample embedded in epoxy resin (Agar Scientific, AGR1030). The prepared sections were stained with alcoholic uranyl acetate and alkaline lead citrate, and examination with Electron transmission electron microscope (FEI Tecnai G2 20 TWIN, USA).

### 2.8 Data analysis

All data are presented as mean ± SEM, and statistical analysis was conducted using GraphPad Prism 8.0 (GraphPad Software Inc., La Jolla, CA, USA) and SPSS (IBM Corp., Armonk, NY, USA) software. The statistical significance was determined using two‐tailed, unpaired Student's *t* tests when comparing two groups, and two‐way ANOVA multiple comparisons test was used when two factors were involved. *p* < 0.05 was considered to be statistically significant.

## 3 RESULTS

### 3.1 Bisulfite sequencing PCR indicated an increase of CpG site methylation of the GJB2 gene promoter region in aging C57/6 J mice

**FIGURE 1:**
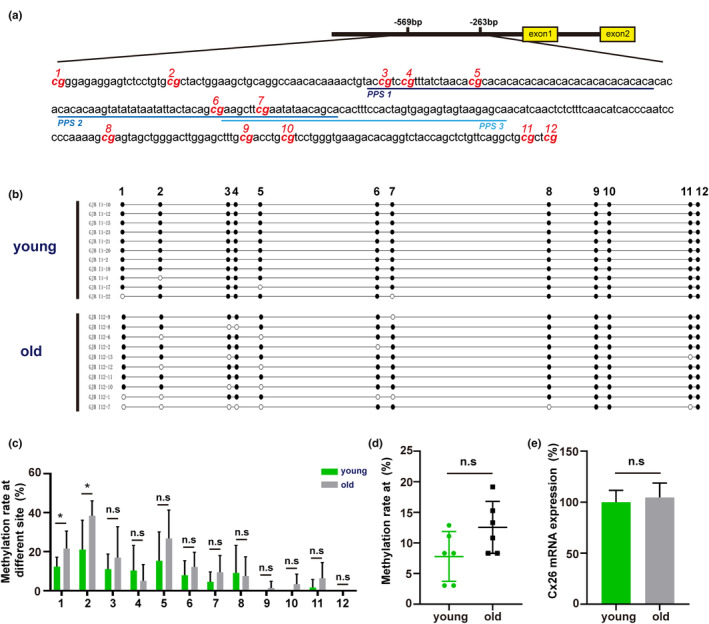
Increase of specific CpG site methylation of the promoter region of GJB2 gene in aging C57/B6 mice. (a) Sequence of target CpG fragments of the promoter region of the C57/B6 mice GJB2 gene. (b) Representative sequencing diagrams of young and aging group. (c) Quantification of the methylation rate at specific CpG sites. (d). Quantification of the total methylated CpG rates of the targeted fragment. (e) Quantification of the mRNA expression level of GJB2 gene. * *p* < 0.05. *n* = 6 mice in each group.

DNA methylation changes dramatically during development and normal aging. Methylation of cytosine residues at CpG sites in the promoter region is involved in the regulation of downstream gene transcription. Here, we evaluated the level of CpG methylation in the promoter region of the GJB2 gene and mRNA levels in the inner ear of aging C57/6J mice. The CG pairs and sequence of fragments are shown in Figure 1a, showing that the GJB2 gene contains three promoters and 12 CpG sites. The results of bisulfite sequencing analysis of the GJB2 gene are shown in Figure 1b. Quantitative results showed that specific CpG sites in the promoter region of the GJB2 gene were hypermethylated in aged C57/6J mice, as compared with younger mice (Figure 1c). However, there was no significant difference in the overall methylation rate (Figure 1d). Moreover, the mRNA expression levels of GJB2 were measured by quantitative RT‐PCR (qRT‐PCR). As can be seen in Figure 1e, mRNA expression levels of the GJB2 gene did not significantly differ between young and old C57/6J mice.

### 3.2 Reduction of Cx26 expression and changes in GJPs during aging

**FIGURE 2:**
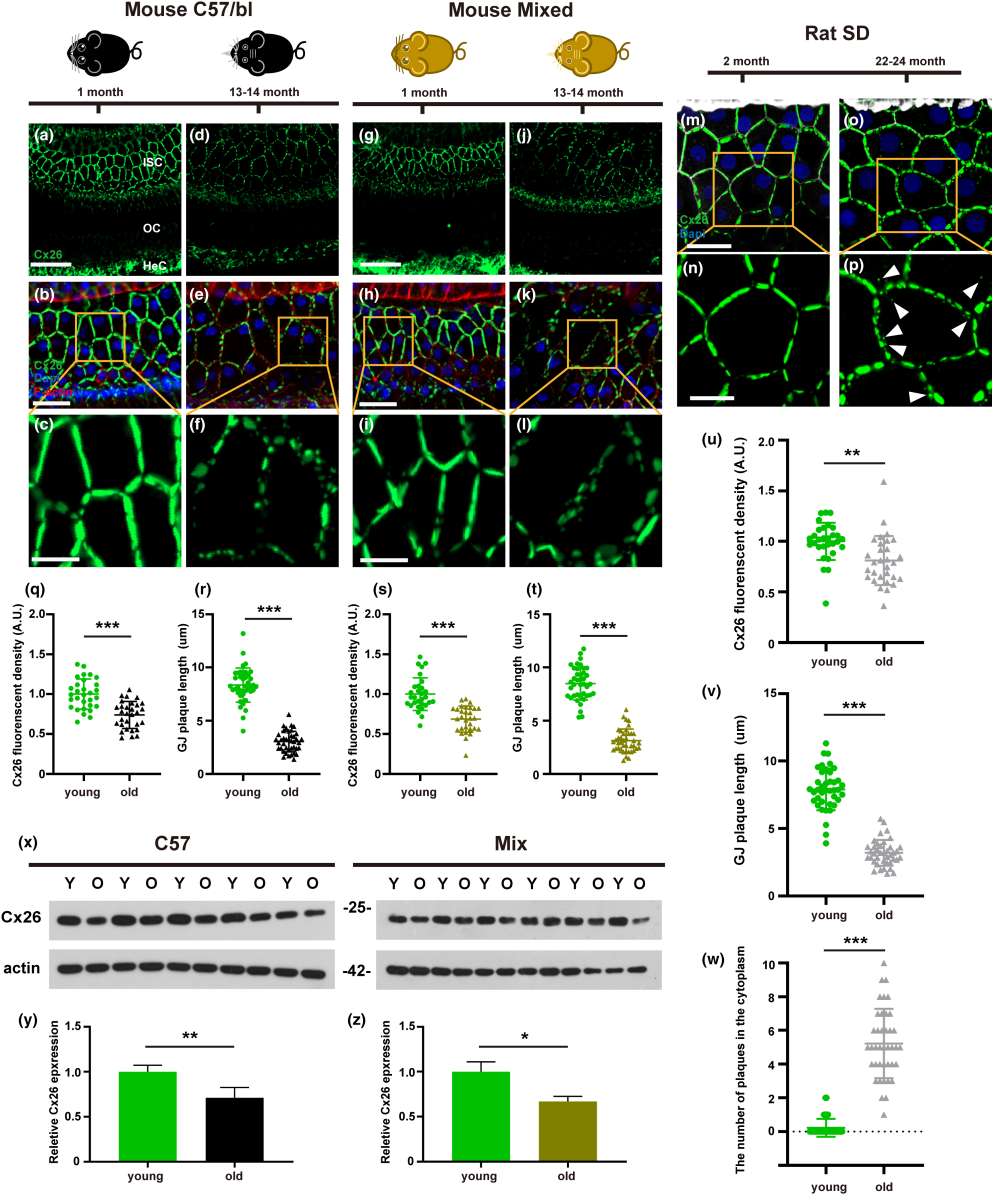
Reduction of Cx26 expression and changes in GJPs during aging. (a–c) Representative images of Cx26 in cochlear ISCs from 1‐month‐old C57/B6 mice. (d–f) Representative images of Cx26 in cochlear ISCs from 13‐month‐old C57/B6 mice. (g–l) Images of Cx26 of ISCs from young and old mixed strain mice. (m–p) Representative images of Cx26 in cochlear ISCs from young and old rat. (q, r) Quantification of the relative fluorescence intensity and length of GJP along a single cell border of ISCs from young and old C57/B6 mice. (s, t) Quantification of the relative fluorescence intensity and length of GJP along a single cell border of ISCs from young and old mixed strain mice. (u–w) Quantification of the relative fluorescence intensity, length of GJP, and the number of Cx26 plaques along a single cell border of ISCs from young and old rats. (x) Total amount of cochlear Cx26 protein levels were assessed in different group from C57/B6 and mixed strain mice by western blotting. (y, z) Quantification of the Cx26 protein levels by normalized to β‐actin. *p < 0.05, **p < 0.01, ***p < 0.001. Scale bars: 40 μm (panels a, g), 20 μm (panels b, h, m), and10 μm (panels c, i, n).

To investigate age‐related changes in gap junctions in the inner ear, we performed immunofluorescent staining to analyze the changes in GJPs of specific supporting cells. In addition, western blotting was used to detect changes in Cx26 expression. In C57/6J mice, GJPs formed linear structures along adjacent cell membranes of inner sulcus cells (ISCs) in the young group (Figure 2a–c). In contrast, GJPs of ISCs in the old group appeared drastically fragmented and as small spots around the cell–cell junction sites (Figure 2d–f). Quantitative immunofluorescence results showed that Cx26 expression in the boundary of the ISCs decreased significantly in the old group (Figure 2q). The average length of GJPs of ISCs were significantly shorter in the old group than in the young group (Figure 2r). Similarly, we observed almost the same phenomenon in mixed strain mice obtained by crossbreeding the C57/6J mice with CBA mice (Figure 2g–l). Quantitative results also showed that Cx26 expression and length of the GJPs of ISCs were significantly decreased in the mixed strain (Figure 2s,t). To further assess this reduction, western blotting was performed to determine the expression of Cx26 protein in the cochlea. Western blot results also confirmed that Cx26 protein levels in the cochleae of the old group were significantly decreased both in C57/6J and in mixed strain mice (Figure 2x–z).

Moreover, we also observed the disruption of GJPs in ISCs with age in rats (Figure 2m–p). The GJPs in the ISCs of the aged rats were beaded, and the number of GJPs in each edge of the ISCs in the aged rats significantly increased (Figure 2w). Quantitative results showed that Cx26 expression and length of GJPs of ISCs in rats were both significantly reduced compared with young group (Figure 2u,v).

### 3.3 Cx26 protein in the cochlea degrades in a variety of ways with aging

**FIGURE 3:**
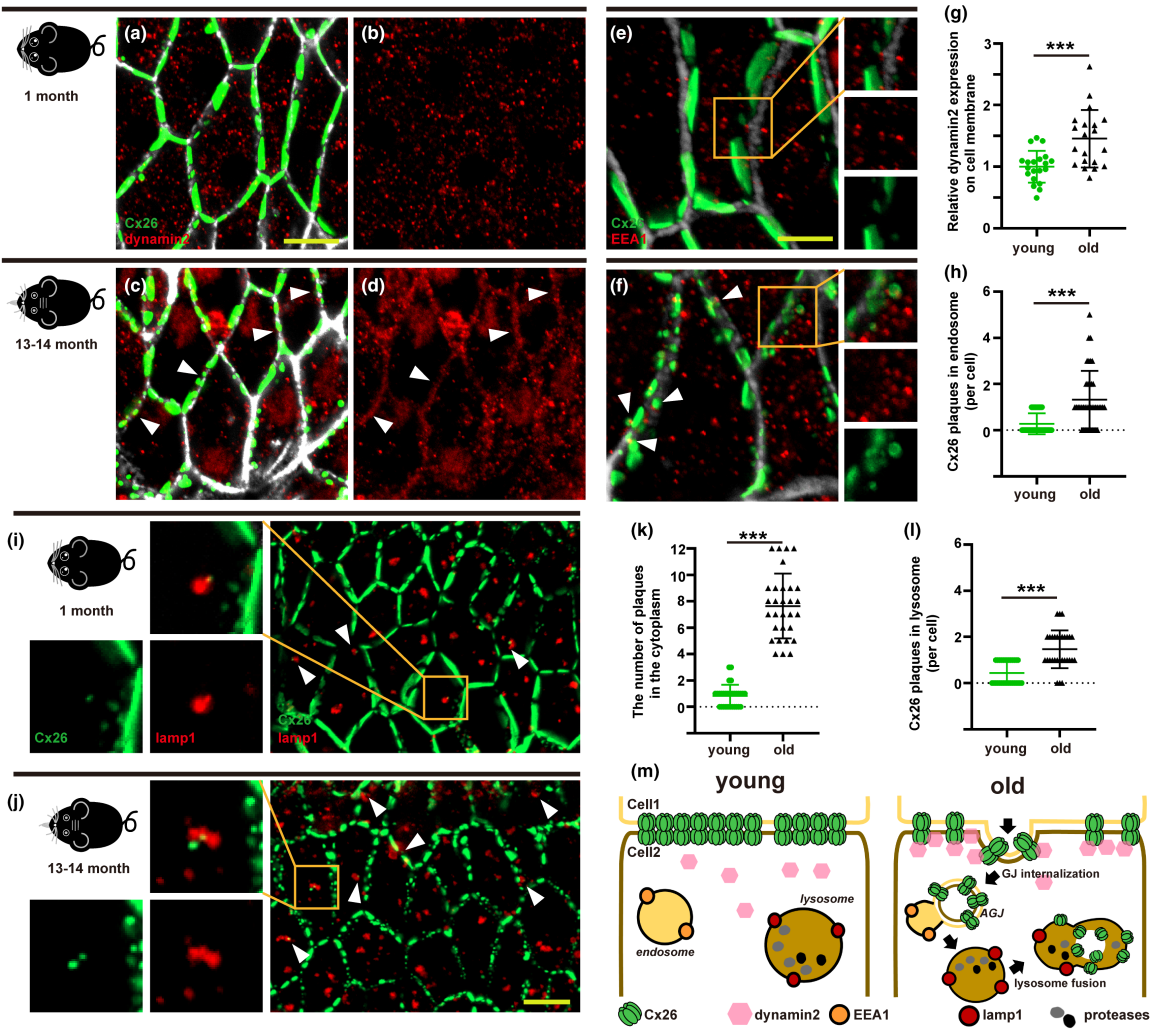
Cx26 protein in cochlea degrades in a variety of ways with aging. (a–d) Representative images of co‐staining with EEA1 and Cx26 in cochlear ISCs from 1‐month and 13‐month‐old C57/B6 mice. (e, f) Endosome were stained using EEA1(red), and counterstained with Cx26 (green) from 1‐month and 13‐month‐old C57/B6 mice. (g) Quantification of the relative fluorescence intensity of dynamin 2 of ISCs from 1‐month and 13‐month‐old C57/B6 mice. (h) Quantification of the number of Cx26 plaques in the endosomes. (i, j) Lysosome were stained using LAMP‐1 (red), and counterstained with Cx26 (green) from different group. (k, l) Quantification of the number of Cx26 plaques in the cytoplasm and lysosome in C57/B6 mice. (m) Schematic diagram of Cx26 degradation process. ***p < 0.001. Scale bars: 10 μm.

Gap junction protein expression and degradation is a unique physiological process, and the specific molecular mechanism remains unclear. Previous studies have reported that gap junction plaques degrade through multiple pathways, involving proteasomes, lysosomes, or autophagosomes (Carette et al., 2015). Gap junction plaques are internalized by endocytosis prior to degradation. Xiao et al. (2014) reported that the interaction between Dynamin 2 (Dyn2) and gap junction protein is particularly important in regulating the endocytosis pathway. As shown in Figure 3a, GJPs formed linear structures along adjacent cell membranes of ISCs and Dyn2 expression was almost absent at cell boundaries in the young group. However, GJPs of ISCs in the old group appeared drastically fragmented and Dyn2 expression was significantly increased at the cell boundary (Figure 3c,d). In addition, we observed that endosomes are abundant in ISC cells in the cochlea of aged mice, and some endosomes are distributed around fragmented gap junctions. Partially internalized Cx26 close vicinity to the endosome, or even partially contacted (Figure 3e,f), which may suggest that the fragmented Cx26 plaque enters the cell through the internalization process. Quantitative results showed that the number of Cx26 plaques in the endosomes was significantly increased in the old group (Figure 3h). Furthermore, LAMP1 was used to label lysosomes and co‐stain with Cx26. The number of Cx26 plaques in the cytoplasm of the old group was significantly increased in C57/6J mice (Figure 3k). Co‐staining results showed that some of the Cx26 plaques entering the cytoplasm co‐localized with lysosomes, suggesting that Cx26 plaques may be finally degraded by the lysosomal pathway (Figure 3i,j). Quantitative results showed that the number of Cx26 plaques in lysosomes was significantly increased in the old group (Figure 3l).

### 3.4 Accumulation of reactive oxygen species (ROS) results in a decrease of Cx26 expression in cochlear explant cultures

**FIGURE 4:**
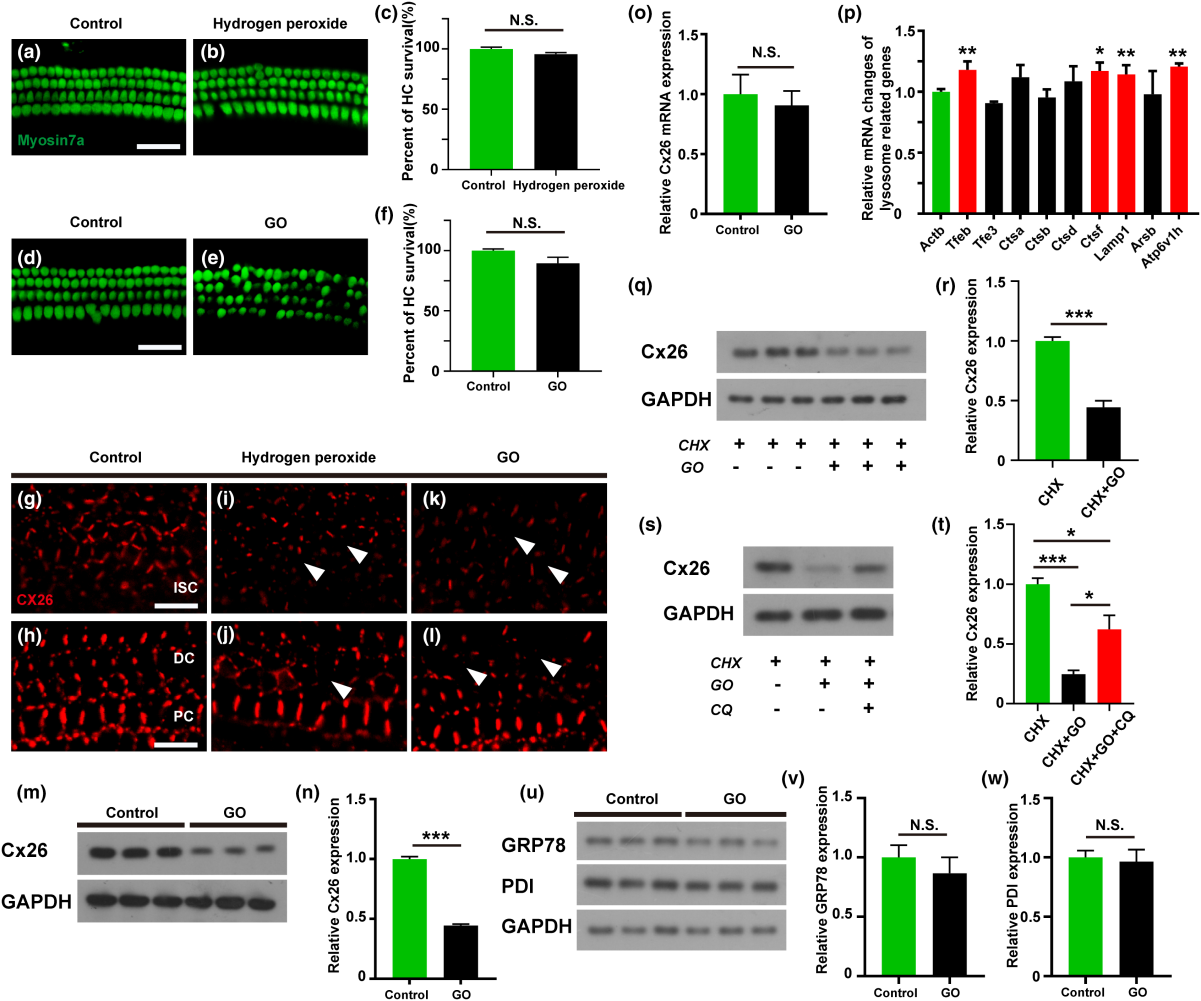
The accumulation of ROS results in the decrease of Cx26 expression in cochlear explant culture. (a–f) Representative images of HCs (green) from hydrogen peroxide or GO group, and quantified the number of HCs. (g–l) Representative images of Cx26 in cochlear DCs from hydrogen peroxide or GO group. (m) Total amount of cochlear Cx26 protein levels following treated with GO were assessed by western blotting. (n) Quantitative expression of Cx26 in cochlea after treated with GO. (o) Quantification of the mRNA expression in cochlea after treated with GO. (p) Characterization of lysosome‐related genes expression changes in the cochlea after treated with GO. (q, r) Total amount of cochlear Cx26 protein levels following treated with GO were assessed by western blotting, and quantitative expression of Cx26. (s, t) Effects of chloroquine on Cx26 degradation induced by GO. (u–w) The expression levels of ER stress‐related proteins GRP78 and PDI in cochlea were assessed by western blotting. *p < 0.05, **p < 0.01, ***p < 0.001. Scale bars: 20 μm.

Previous studies have shown that the accumulation of ROS in the cochlea is one of the important causes of ARHL. In cochlear explants, we constructed ROS models induced by hydrogen peroxide or glucose oxidase (GO). Treatment with 500 μmol/L hydrogen peroxide or GO at a concentration of 60 U/L for 4 h had little effect on hair cell survival (Figure 4a–f). In the apical turns of control group, GJPs presented a linear structure distributed between adjacent cell membranes of ISCs (Figure 4g). In contrast, GJPs of ISCs in the apical turns of hydrogen peroxide or GO groups showed drastically smaller‐sized spots around the cell–cell junction sites and weak fluorescence signals (Figure 4i,k). In addition, we also observed the narrowing or partial absence of GJPs in Deiter's cells following treatment with hydrogen peroxide or GO (Figure 4h–l). The levels of Cx26 protein was significantly decreased in the GO group (Figure 4m,n). In addition, quantitative PCR results showed that the transcription levels of Cx26 did not significantly changed in the GO treatment groups (Figure 4o). Taken together, these results suggested that accumulation of ROS leads to Cx26 protein degradation and GJP destruction in cochleae. To confirm that ROS accumulation causes Cx26 degradation, cochlear explants were treated with CHX to inhibit protein synthesis. Treatment with GO caused a decreased in Cx26 expression in cochlear explants in the presence of CHX, which indicated that the accumulation of ROS induced by GO enhanced the degradation of Cx26 (Figure 4q,r). Moreover, we examined the mRNA expression levels of a series of lysosome‐related genes, and the transcription levels of TFEB, CTSF, LAMP1 and ATP6V1H genes increased in the GO group, indicating lysosome hyperfunction (Figure 4p). Treatment with the lysosomal inhibitor chloroquine significantly reversed the degradation of Cx26 induced by GO (Figure 4s,t). There was no significant change in the expression of the ER stress‐related proteins GRP78 and PDI in the GO group, suggesting that ER stress was not involved in the Cx26 degradation induced by GO (Figure 4u–w).

### 3.5 Distinct patterns of hair cell loss in the different targeted‐cell Cx26‐null mouse model

**FIGURE 5:**
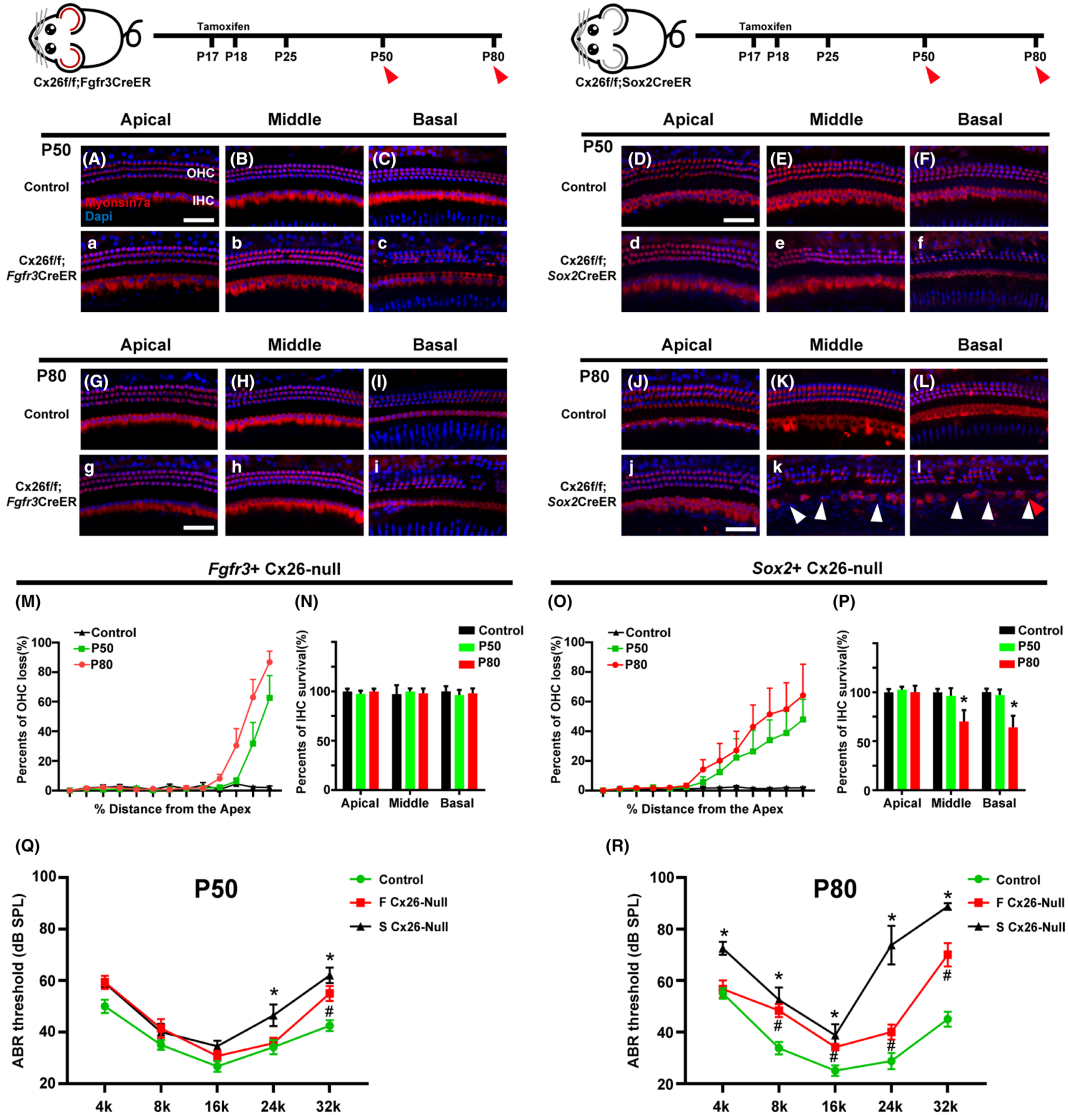
Distinct hair cell loss patterns and hearing loss in the different Cx26‐null mouse model. (A–C, a–c) Representative images of HCs (Myosin7a, red) of different turns in control (A–C) and Fgfr3‐Cx26 null group (a–c) at P50. (D–F, d–f) Representative images of HCs of different turns in control and Sox2‐Cx26 null group at P50. (G–L, g–l) Representative images of HCs in control, Fgfr3‐Cx26 null or Sox2‐Cx26 null group at P80. (M–P) OHCs or IHCs count in the Fgfr3‐Cx26 null and Sox2‐Cx26 null group. (Q, R) ABRs were measured in control and different Cx26‐null group *p < 0.05. Scale bars: 40 μm.

Two distinct Cx26‐null mouse models were established to explore whether degradation of Cx26 in the cochlea is directly related to ARHL. In the Cx26‐null group, Cx26 of DCs and PCs was knocked out successfully in both the Fgfr3‐iCreERT2 and Sox2CreER cell lines. At P50, a significant cellular degeneration of outer hair cells (OHCs) was observed in the basal turn of the Cx26^f/f^; Fgfr3‐iCreERT2 mice (Figure 5C). At P80, OHC loss was increased in the basal turn (Figure 5I). Moreover, no substantial inner hair cell (IHC) loss was found in any turns of the Cx26^f/f^; Fgfr3‐iCreERT2 mice (Figure 5G–I). Quantification of OHCs and IHCs of the Cx26^f/f^; Fgfr3‐iCreERT2 line are shown in Figure 5M,N. Similarly, OHCs loss was also observed in the middle and basal turn of the Cx26^f/f^; Sox2CreER mice at P50 (Figure 5E,F). No substantial IHC loss was found in any turns of the Cx26^f/f^; Sox2CreER mice at P50. However, at P80, there was a significant cellular degeneration of IHCs in the Cx26f/f; Sox2CreER mice (Figure 5K,L). Quantification of OHCs and IHCs in all turns of the Cx26^f/f^; Sox2CreER line are shown in Figure 5O,P. We performed ABR tests at P50 and P80, using the littermates without Cre as controls. These two lines showed specific high‐frequency hearing loss. At P50, the auditory threshold of the Cx26^f/f^; Fgfr3‐iCreERT2 line was significantly increased at 32 kHz, and the Cx26^f/f^; Sox2CreER line showed 13 dB and 20 dB auditory threshold shifts at 24 kHz and 32 kHz (Figure 5Q). At P80, hearing loss was further aggravated and extended to other frequencies. Almost all frequencies were affected, and the high‐frequency hearing loss was still more serious (Figure 5R).

### 3.6 Demyelination and degeneration of spiral ganglion neurons (SGNs) were observed in knockout mice

**FIGURE 6:**
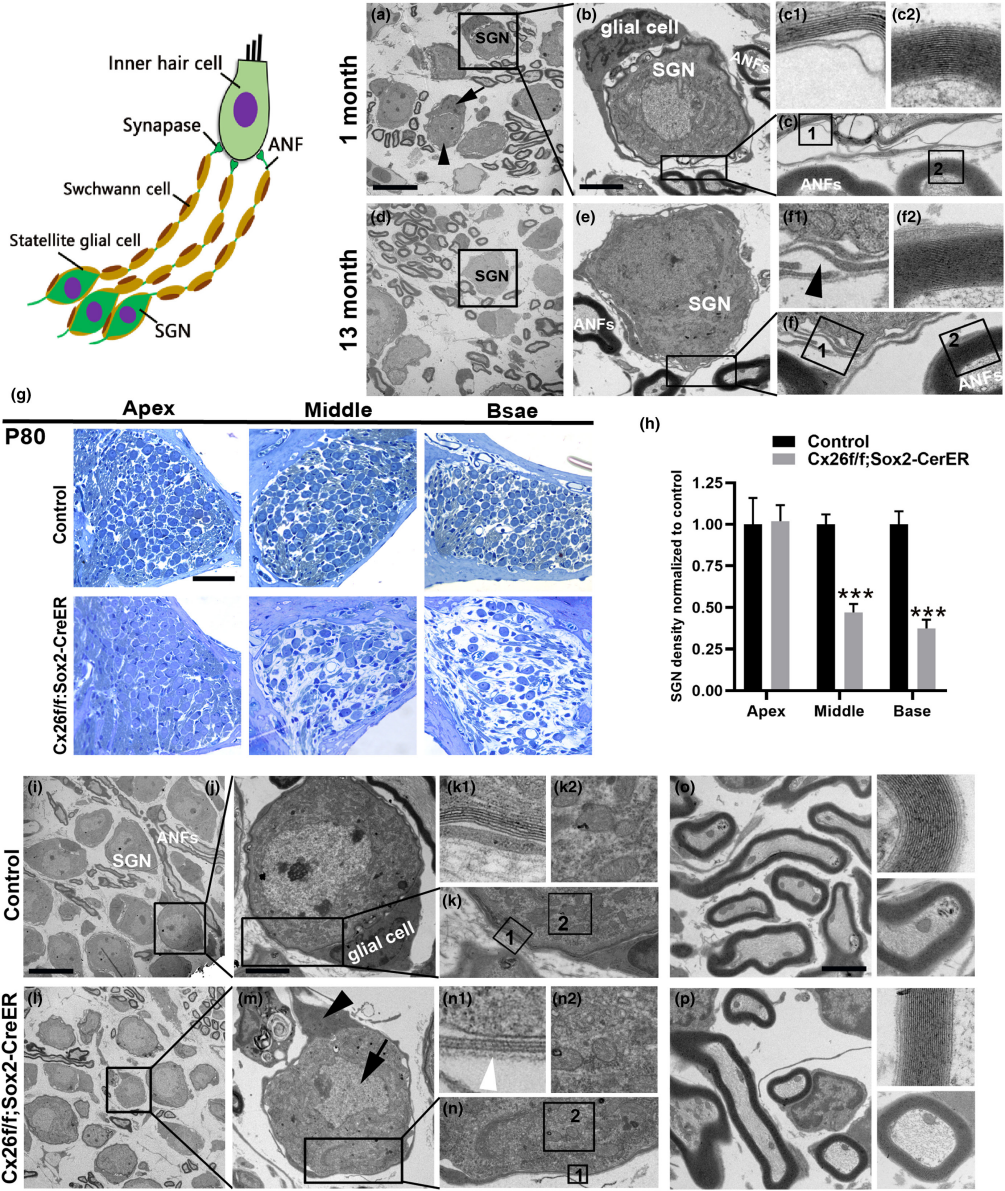
Demyelination and degeneration of spiral ganglion neurons (SGNs) were observed in Cx26‐null mice. (A–C) The ultrastructural morphology of SGNs in the 1‐ month‐old C57BL/6J mice. (D–F) Demyelination and degeneration of SGNs were observed in aged C57BL/6J mice. (G) Representative images of SGNs from the control group and Sox2‐Cx26null group at P80. (H) Quantification of the survival of SGNs. (I–P) The ultrastructural morphology of SGNs and ANF in the control group or Sox2‐Cx26null group. The black arrows and arrowheads indicate the SGNS and satellite glial cell. The white arrow indicates the abnormal myelin structure of SGNs in the Cx26 null group. ***p < 0.001. Scale bars: 10 μm (panels A, I), 30 μm (panels G), and 4 μm (panels B, J, O).

Cochlear auditory nerve fibers and SGNs are encased in myelin sheath to provide insulation and ensure precise transmission of nerve impulses. Demyelination of SGNs or nerve fibers is a common pathogenesis of ARHL (Bao & Ohlemiller, 2010). We examined the ultrastructure of SGNs in aged mice and observed degeneration of SGNs. Electron microscopic images of young mice showed that SGNs were ensheathed within multiple layers of compact myelin, and other subcellular structures appeared normal (Figure 6A–C). In aged mice, the myelin sheath of SGNs was disordered and the multilayer compact sheath structure of myelin was lost (Figure 6D–F). Furthermore, we also analyzed the structural changes of SGNs in Cx26‐null mice. Observation of radial sections showed obvious degeneration of SGN cells in Cx26‐null mice (Figure 6G,H). In the control group, the ultrastructure of SGNs and nerve fibers was normal (Figure 6J,K,O). The ultrastructural changes of the SGNs in Cx26‐null mice were similar to those in naturally‐aged mice. In the Cx26‐null group, we observed significant structural changes of SGNs and even loss of glial cells in some SGNs (Figure 6L). As shown in Figure 6M, the nuclei of SGNs were shriveled. In addition, the number of layers of myelin and the thickness of the myelin sheaths were significantly reduced compared with those of the control group (Figure 6N). The ultrastructure of nerve fibers in the Cx26‐null group was not affected (Figure 6P).

## 4 DISCUSSION

### 4.1 Disrupted GJPs in the cochleae of aged C57BL/6J mice

The C57BL/6J mouse strain is considered an ideal animal model for the study of ARHL because hearing loss occurs at 6 months of age and then progressively worsens (Kane et al., 2012; Miyasaka et al., 2013). We validated the ABR threshold in C57BL/6J mice and found significant high‐frequency hearing loss at 6 months and hearing impairment at all frequencies at 12 months (data not shown). We analyzed transcription of the GJB2 gene in the cochleae of young and old C57BL/6J mice, and found that the mRNA level of the GJB2 gene was not significantly altered in old mice compared with young mice. Our bisulfite sequencing analysis showed that specific CpG sites in the promoter region of the GJB2 gene were hypermethylated in aged mice, but overall methylation rates were not significantly different. Although our previous studies revealed that the expression of GJB2 and hypermethylation of its DNA promoter in the inner ear of mimetic aging rats were decreased (Wu et al., 2014), this difference in results may be due to species differences or different forms of aging. In addition, western blot results showed that the total amount of Cx26 protein in the cochleae of aged mice was significantly reduced. Taken together, we can conclude from these results that the decrease in the protein levels of Cx26 was not caused by a decrease in GJB2 gene transcription, but rather due to reduced Cx26 protein synthesis or increased post‐synthesis degradation.

To further clarify the changes of Cx26 protein in the cochleae of aged mice, we performed immunofluorescence staining and analyzed the GJPs in ISCs. GJPs of ISCs in aged mice appeared drastically fragmented with small spots around the cell–cell junction sites. The GJP sizes and Cx26 expression of ISCs were both significantly reduced. These data suggest that the dysfunction or degradation of some key proteins that maintain cochlear function may be the direct cause of ARHL and may even extend to other age‐related diseases.

Connexin is a short‐cycle protein with a half‐life of 1.5–5 h (Falk et al., 2014). The synthesis and degradation of connexins are balanced under physiological conditions. Gap junction channels that perform functions between cells are constantly renewed, and old channels enter the cell by endocytosis for subsequent degradation in lysosomes (Totland et al., 2020). Several studies have shown that cells can modulate the rate of connexin degradation in response to various pathophysiological changes (Johnson et al., 2013; Leithe & Rivedal, 2004; Rivedal & Leithe, 2005). In our study, we investigated Cx26 degradation in the cochleae of aged mice. The expression of Dyn2, a protein that mediates internalization, was increased in the membrane of ISCs from aged mice. In addition, we observed the co‐localization of Cx26 with EEA‐1 or LAMP‐1, suggesting that Cx26 enters endosomes or lysosomes to further complete degradation after internalization. Kamiya et al. (2014) reported failure to form complete GJPs in the Cx26^R75W+^ mouse model, and fragmentation of Cx26 plaques was associated with excess endocytosis and increased caveolin expression. Our previous studies also reported that the normal development of Cx26 in supporting cells is essential for OHC survival and cellular function (Chen, Xu, et al., 2018). Currently, whether there is a direct relationship between gap junction plaque destruction and ARHL is unclear. The decrease in cochlear gap connexins and the destruction of GJPs can be regarded as a landmark event in ARHL.

### 4.2 The degradation of Cx26 in the cochleae of aged mice may be related to ROS accumulation

The formation of cellular GJPs is an extremely complex physiological process that includes translation, posttranslational modification, transport, and assembly at the plasma membrane (Totland et al., 2020). After translation, connexins enter the endoplasmic reticulum (ER) for post‐translational modification. During this process, some of the connexin undergoes ER‐related degradation, while the remaining undegraded connexin is transported to the Golgi for further processing and then transported to the cell membrane in the form of vesicles (Mitra et al., 2006; Shaw et al., 2007). Previous studies have demonstrated that ROS accumulation causes oxidation damaged to major macromolecules, such as lipids, proteins, and DNA, leading to cell damage (Rigoulet et al., 2011). Moreover, increased production of ROS has been reported to affect connexin expression and to be associated with changes in the function of GJs (Martínez & Sáez, 2000). Whether the degradation of Cx26 plaques in the cochleae of aged mice is related to ROS accumulation remains to be investigated. Previous studies have shown that ROS accumulation and reduced antioxidant capacity play an important role in age‐related diseases, including ARHL (Davalli et al., 2016; Rousset et al., 2020). Our in vitro experiments revealed that both exogenous or endogenous ROS accumulation led to a decrease of Cx26 expression. In the cochlear explant culture model, we observed that ROS accumulation caused Cx26 degradation and GJP disruption, accompanied by increased expression of lysosome‐related genes. Treatment with the lysosomal inhibitor chloroquine significantly reversed the degradation of Cx26 induced by ROS accumulation. In the cochlea of aged mice, intracellular ROS accumulation may be the main factor initiating the degradation of GJPs, and the lysosomal pathway is involved in the degradation process of GJPs. It is recognized that the accumulation of ROS in the cochlea may cause DNA damage or mitochondrial disorder and subsequent sensory cell or SGN damage, which is the main cause of ARHL. Here, we propose that ROS accumulation may trigger the degradation of important proteins in the cochlea, which should be further studied and may be one of the main contributors to ARHL.

### 4.3 Reduction of Cx26 expression in adult mice results in a pathological phenotype similar to ARHL

In the clinic, mutations in Cx26 cause various auditory phenotypes ranging from profound congenital deafness to late‐onset progressive hearing loss (Chan & Chang, 2014; del Castillo & del Castillo, 2011). A series of Cx26‐knockout or mutant animal models also exhibit similar auditory phenotypes. Studies have shown that a variety of pathological changes are involved in hearing loss caused by Cx26 deficiency. Our previous study demonstrated that developmental disorders of pilar cell (PCs) may be the main cause of severe hearing loss in Cx26‐deficient mice (Chen et al., 2014; Chen, Xie, et al., 2018). In addition, there are Cx26‐knockout mice that show late‐onset hearing loss, in which the late‐onset hearing loss results from reduction of active cochlear amplification (Zhu et al., 2015). Fetoni et al. (2018) constructed a Gjb2^+/−^ mouse model to simulate the heterozygous population carrying Gjb2 gene mutations, and showed that Gjb2^+/−^ mice exhibited high‐frequency hearing loss and a decline in distortion product otoacoustic emissions over time. This study is the first to link a deficiency or absence of Cx26 to ARHL. Furthermore, Tajima et al. (2020) observed that Cx26 degradation occurs before hair cell death in C57BL/6J mice, which suggests that degradation of Cx26 and disruption of GJPs is involved in the early development of ARHL. Currently, there is no evidence that Cx26 depletion or GJP disruption is directly related to ARHL. We have systematically explored the expression and distribution of gap junction proteins in mouse cochlea. Cochlear basilar membrane Deiters' cells (DCs) correspond to outer hair cells one by one. Combined with our previous studies, the expression of Cx26 in supporting cells is essential for maintaining the survival of outer hair cells (Chen, Xu, et al., 2018). Furthermore, we observed a significant decrease in Cx26 expression and drastically smaller‐sized spots around the cell–cell junction sites in DCs and PCs in aged mice. Therefore, we speculate that the Cx26 of basilar membrane supporting cells may be directly related to the development of age‐related hearing loss. Here, we established two conditional knockout models to knock down Cx26 in supporting cells of adult mice, mimicking the reduced Cx26 expression in aged mice. ABR results showed that both models exhibited high‐frequency hearing loss, which gradually progressed over time. Pathological examination showed that the degeneration of OHCs and SGNs were observed in Cx26‐knockdown mice. The degeneration of OHCs occurred in the basal turns and spread to the middle turns over time. Our data suggest that premature reduction of Cx26 expression in adult mice results in a pathological phenotype similar to ARHL. Therefore, we can conclude that the degradation of Cx26 in the cochlea accelerates the occurrence of ARHL.

In summary, our results support the hypothesis that reduction of Cx26 and disruption of GJPs in the cochlea contribute to the development and progression of ARHL. The degradation of Cx26 tends to occur in the early stages of ARHL. Therefore, it can be used as an early warning marker of ARHL. Furthermore, treatment targeting Cx26, such as GJB2 gene therapy, may be effective for ARHL.

## AUTHOR CONTRIBUTIONS

All authors contributed to the study's conception and design. Yu Sun and Wei‐jia Kong conceived and designed the experiments; Kai Xu, Sen Chen, Xiao‐zhou Liu, and Xiao‐hui Wang performed the experiments; Xue Bai, Le Xie, and Yue Qiu analyzed the data; Kai Xu and Sen Chen wrote the paper. Yu Sun and Wei‐jia Kong reviewed and edited the manuscript. All authors read and approved the final manuscript.

## CONFLICT OF INTEREST STATEMENT

The authors declare no conflict of interest.

## Data Availability

All data that support the findings in this study are available from the corresponding author upon reasonable request.
